# [^18^F]FDG metabolic brain network in C58/J strain: an autism murine model

**DOI:** 10.1007/s00429-026-03087-8

**Published:** 2026-03-04

**Authors:** Leticia Verdugo-Díaz, Antonieta Martínez-Guerrero, Diana Cecilia García-García, Arturo Avedaño-Estrada, Miguel Ángel Ávila-Rodríguez, Daniel Garzón-Cortés, Mónica Martínez-Marcial, Valeria Canuto-Ramírez, Gabriel Roldán-Roldán, Elizabeth Ibarra-Coronado

**Affiliations:** 1 Laboratorio de Bioelectromagnetismo, Departamento de Fisiología, Facultad de Medicina, Universidad Nacional Autónoma de México, Ciudad de Mexico, México; 2 Laboratorio de Sistemas Complejos, Centro de Investigación en Ciencias, Universidad Autónoma del Estado de Morelos, Cuernavaca, Morelos México; 3 Licenciatura en Fisica Biomédica, Facultad de Ciencias, Universidad Nacional Autónoma de México, Ciudad de Mexico, México; 4 División de Investigación, Facultad de Medicina, Unidad Radiofarmacia-Ciclotrón, Universidad Nacional Autónoma de México, Ciudad de Mexico, México; 5 Centro de Investigación sobre el Envejecimiento, Centro de Investigación y de Estudios Avanzados Sede Sur, Ciudad de Mexico, México; 6 Unidad de Modelos Biológicos, Instituto de Investigaciones Biomédicas, Universidad Nacional Autónoma de México, Ciudad de Mexico, México; 7 Programa de Apoyo y Fomento a la Investigación Estudiantil (AFINES), Facultad de Medicina, Universidad Nacional Autónoma de México, Ciudad de Mexico, México; 8 Laboratorio de Neurobiología Conductual, Departamento de Fisiología, Facultad de Medicina, Universidad Nacional Autónoma de México, Ciudad de Mexico, México; 9 Centro de Ciencias de la Complejidad, Universidad Nacional Autónoma de México, Ciudad de México, Mexico

**Keywords:** Autism spectrum disorder, Behavior, Metabolic brain network, C/58 J strain, Positron emission tomography (PET)

## Abstract

Autism spectrum disorder (ASD) is associated with atypical brain network organization. Functional connectivity has been extensively studied using fMRI, which often reports reduced long-range connectivity alongside local hyperconnectivity. However, BOLD-based connectivity reflects neurovascular coupling and therefore provides an indirect estimate of neuronal activity. Complementary information may be obtained from [^18^F]FDG microPET, which quantifies regional glucose uptake—an index of synaptic energy demand integrated over minutes—although FDG-derived network organization has been scarcely examined in ASD. Here, “metabolic connectivity” refers to between-subject covariance in regional [^18^F]FDG uptake (group-level metabolic covariance networks), not within-subject temporal coupling as in BOLD-fMRI functional connectivity. We tested whether the C58/J mouse strain—an ASD-relevant model with social deficits and repetitive behaviors—recapitulates ASD-relevant metabolic network-level alterations. Using [^18^F]FDG microPET, we constructed ROI-wise metabolic covariance networks by correlating uptake values across animals and compared C58/J mice with C57BL/6 controls. The C58/J network exhibited higher clustering and a more locally cohesive organization than the C57BL/6 network. Nodal degree/edge density was reduced in the olfactory bulb, hippocampus, and hypothalamus. In contrast, the motor-related regions—including the striatum, brainstem, and superior and inferior colliculi—showed a higher degree/denser covariance. These findings suggest that C58/J mice display FDG-derived metabolic covariance network features qualitatively consistent with those reported in ASD FDG-PET studies, supporting this strain as a tool to investigate ASD-relevant pathophysiological mechanisms and to evaluate candidate interventions.

**Supplementary Information:**

The online version contains supplementary material available at 10.1007/s00429-026-03087-8.

## Introduction

Autism spectrum disorder (ASD) is a neurodevelopmental disorder (NDD) that encompasses a spectrum of severe and heterogeneous neuropsychological impairments that manifest across an individual’s developmental trajectory. Clinically, ASD is associated with social-communication difficulties and restricted and repetitive behaviors (American Psychiatric Association 2013); however, the neurobiological mechanisms that contribute to these phenotypes remain incompletely understood. In this context, neuroimaging studies have contributed to the understanding of ASD neurobiology by characterizing alterations in large-scale brain organization and by providing a framework to relate brain alterations to symptomatology (Dawson et al. 2023).

The ASD etiology is not clear; diagnosis is currently based on behavioral criteria, and symptoms typically emerging in early childhood often co-occur with atypical sensory processing and language delays, reflecting the heterogeneity of its presentation (American Psychiatric Association 2013). Recent surveillance indicates a high and increasing prevalence of ASD, underscoring the need to clarify its neurobiological mechanisms and to develop effective interventions (Maenner 2021). Although neurobiology is poorly understood, it is known that a complex relationship exists between environmental and genetic factors that likely influence the heterogeneity of its presentation, contributing to the difficulty of diagnosis due to the lack of biomarkers that identify this condition. A large number of studies have provided evidence that the brain in ASD exhibits anatomical and functional alterations, which also show a correlation with the degree of severity (Liu and Huang 2020). In the brain organization and communication, the connectivity analysis is a tool for describing neurophysiological activity in different conditions or cognitive processes (Bullmore and Sporns 2009; Park and Friston 2013) (across development, or in a variety of neurological diseases). The temporal coincidence of these neurophysiological events, which may or may not be anatomically close, defines functional connectivity (Friston 1994). In this context, the connectivity pattern results from a statistical relationship between the measures of physiological activity recorded in different areas (Bassett and Sporns 2017).

Neurophysiological activity can be measured through different indicators such as electroencephalographic (EEG), magnetoencephalographic (MEG), functional magnetic resonance imaging (fMRI) or positron emission tomography (PET) recordings. Studies of ASD functional connectivity using task-based and resting-state fMRI and EEG have frequently revealed altered brain organization particularly at the cortical level (Liu and Huang 2020; Dawson et al. 2023). Specifically in the fMRI studies report hypoconnectivity during tasks involving language(Just et al. 2004), working memory (Koshino et al. 2005), and imagination (Kana et al. 2006), while some research has identified hyperconnectivity in areas related to visuomotor processing (Mizuno et al. 2006), emotional regulation (Welchew et al. 2005), and some aspects of language (Shih et al. 2010). The functional connectivity in fMRI based on BOLD signal measures changes related to oxygenation in the blood vessels that irrigate a given brain region, in a process dependent on the neurovascular coupling that underlies the hemodynamic response, in an indirect relationship between oxygen consumption and neuronal activity (Sala et al. 2023).

Comparatively the same neural activity elicits stronger responses in glucose metabolism (Fox et al. 1988; Sala et al. 2023), as evidenced through the PET, where [^18^F]FDG is a direct indicator of synaptic activity, as it measures the neurometabolic coupling between glucose and hexokinase (Magistretti and Allaman 2015), the enzyme responsible for glycolytic activity in neurons (Sokoloff 1977). In the study of connectivity through networks, temporal definition constitutes a substantial difference between signals. In the case of the [^18^F]FDG PET signal, a network is built based on inter-subject estimation; that is, connectivity is estimated from the covariation of measurements between subjects, where only one image per individual is available. In contrast, connectivity based on the BOLD signal is evaluated within the subject, where the covariation measures through which the network is constructed belong to different time points of the same subject, obtaining an estimate for each individual. Even though connectivity studied in both modalities indexes neuronal activity, each one reflects different processes; accordingly, it is plausible to estimate metabolic connectivity from [^18^F]FDG PET signal recordings, which are more stationery and time-invariant signals, because they capture stable neuronal activity over a longer period (minutes) and are less dependent on neurovascular coupling, potentially resulting in greater reproducibility. Importantly, metabolic covariance networks should be interpreted as group-level patterns of coordinated regional uptake rather than a direct measure of within-subject temporal synchrony. Within this framework, comparing network topology across modalities can provide complementary information about large-scale brain organization and encourage the investigation of metabolic connectivity in ASD (Yakushev et al. 2017; Sala et al. 2023).

The study of metabolic connectivity based on [^18^F]FDG PET is a field that has been poorly studied in the ASD. The [^18^F]FDG PET neuroimaging studies have reported region-specific alterations in cerebral glucose metabolism—an index of synaptic energy demand integrated over tens of minutes—with evidence of both hypo- and hypermetabolism across cortical and subcortical regions. Hypermetabolism have been described in the hippocampus, occipital and cingulate cortices, and basal ganglia, whereas decreases have been reported in the amygdala, premotor frontal and parietal cortices (Li et al. 2021; Tan et al. 2022). The only study conducted by H. Lee in 2012 (Lee et al. 2012) found that the distribution graph of metabolic network connections in individuals with ASD suggests simultaneous local overconnectivity and global underconnectivity. Compared to the controls, the metabolic networks of ASD maintained weak connectivity between the left inferior prefrontal regions and other brain regions. The authors suggest that this finding could explain some of the behavioral symptoms of ASD. This is consistent with findings observed in functional networks based on fMRI and their relationship to cognitive processes, with the consideration that these relationships in metabolic networks estimate a different processes of the same neuronal activity (Dapretto et al. 2006; Courchesne et al. 2007).

The use of biological models is a valuable strategy for gaining deeper insights into the pathophysiology underlying ASD and for accurately evaluating therapeutic interventions developed for its treatment. Murine models have been widely employed in recent decades to investigate the neurobiological mechanisms of ASD. The *Mus musculus* C58/J strain has been proposed as a model for ASD due to its spontaneous exhibition of behaviors that mirror core symptoms of the disorder, behaviors characterized by repetitive patterns such as vertical hind-limb jumps and backward somersaults, both atypical in form and frequency (Moy et al. 2008; Muehlmann et al. 2012).

A recent study has reported reduced size in brain structures associated with motor function in the C58/J strain (Wilkes et al. 2020). These mice also exhibit decreased sociability and abnormal motor activity, including hyperactivity (Moy et al. 2008). These alterations manifest spontaneously from early development and persist throughout the animal’s lifespan (Muehlmann et al. 2012). Furthermore, C58/J mice exhibited impaired learning abilities (Munn et al. 2011); however, higher-order behaviors (e.g., novel-object exploration, marble burying) require further validation (Moy et al. 2014; Whitehouse et al. 2017).

This study aims to evaluate whether the [^18^F]FDG-derived metabolic covariance network organization of the C58/J strain shows patterns consistent with those reported in ASD FDG-PET studies. Understanding these trait-level network characteristics could provide stronger validation for the use of this strain in future experimental studies, both to explore ASD pathophysiology and to develop novel therapeutic strategies.

## Methods

### Ethics

The protocol used was approved by The Committee on Ethics and Use in Animal Experimentation of the School of Medicine, UNAM (FM/DI/061/2022). The study was done following the guidelines of Mexican regulations (NOM-062-ZOO-1999) and the Guide for the Care and Use of Laboratory Animals of the National Institute of Health (NIH) to ensure compliance with the established international regulations and guidelines.

### Animals

Adult male C58/J and C57BL/6 mice (11 weeks old) were obtained from the Biological Models Unit of the Instituto de Investigaciones Biomédicas at the Universidad Nacional Autónoma de México, originally sourced from The Jackson Laboratory (Bay Harbor, ME, USA). The mice were housed in groups of five per cage (made of polypropylene 32 × 22 × 12 cm) in a temperature-controlled room (22–24 °C) with a 12 h light–dark cycle (lights on at 8:00 h). The animals had ad libitum access to sterilized INVIGO 2018 food and purified water.

For PET imaging, 21 animals from the C58/J group and 19 from the C57BL/6 group were used. Behavioral protocols were conducted with independent groups of 25 and 30 animals each.

### MicroPET-acquisition

For PET acquisition data, all mice were fasted for 8 ± 0.5 h before 2-deoxy-2-[^18^F]fluoro-D-glucose ([18F]FDG) injection. [18F]FDG was administered in the tail vein as a bolus (10 ± 4.2 MBq), 60 min before the scan. Data acquisition lasted 10 min per animal. During the radiopharmaceutical biodistribution study, mice were allowed to move freely to minimize the impact of anesthesia on metabolism. Anesthesia was induced through 5% isoflurane in 95% O₂ administration and maintained with 2–3% isoflurane during the scan. PET imaging was performed using a Focus 120 micro-PET scanner (Siemens/CTI-Concord Microsystems, Knoxville, TN, USA). During the acquisition, respiration, heart rate, and temperature were continuously monitored.

### Pre-processing data

All images were processed using PMOD 3.7 software (PMOD Technologies, Zurich, Switzerland). To locate the different anatomical regions of the mouse brain, the image was normalized to an anatomical MRI and a standardized atlas, M. Mirrione (Mirrione et al. 2007), which consists of 19 brain structures. Before spatial normalization, the images were smoothed with a 1 × 1 × 1 mm^3^ full-width-half-maximum (FWHM) Gaussian kernel. Once the image was normalized to MRI and the atlas, the standardized uptake value (SUV) was obtained for each structure.

A ratio analysis was also made to diminish the individual variability; in this method, each SUV of each brain structure was divided by the brain average SUV of the same individual.

### Metabolic network construction and graph metrics

Because [^18^F]FDG–PET acquisitions were static, “connectivity” was not derived from within-subject time series but from between-subject associations in regional uptake. For each strain, we extracted a single value per ROI (SUV/SUVR; see Preprocessing) for every individual and computed an ROI × ROI Spearman rank-correlation matrix across subjects. Given the non-normal distributions of ROI values, we used two-tailed Spearman’s ρ. For each strain, all M = N(N–1)/2 inter-regional pairs were tested and p-values were adjusted using the Benjamini–Hochberg false discovery rate; an edge was retained only if it satisfied both criteria—FDR-adjusted q < 0.05 and magnitude |ρ|> 0.5—yielding a symmetric, undirected, weighted adjacency matrix.

The resulting networks had nearly identical densities, differing by a single edge (Δδ = 2/[N(N–1)]). To rule out density-driven confounds, we repeated all analyses after exactly matching densities by removing the weakest-|ρ| edge from the denser graph; all graph metrics (degree/distribution, clustering, global and local efficiency, betweenness, modularity) were qualitatively invariant. We therefore report results for the FDR- and magnitude-thresholded networks.

Graph analysis was performed in R (RStudio) using igraph and qgraph. To study the network’s dynamics and topology, the modularity, degree distribution, betweenness centrality, global and local efficiency metrics were obtained as shown in supplementary Table 1.

### Behavior assessment

All behavioral tests were performed under the same conditions during the light period between 8:00 and 12:00 h.

#### Olfactory threshold assessment

**Fig. 1:**
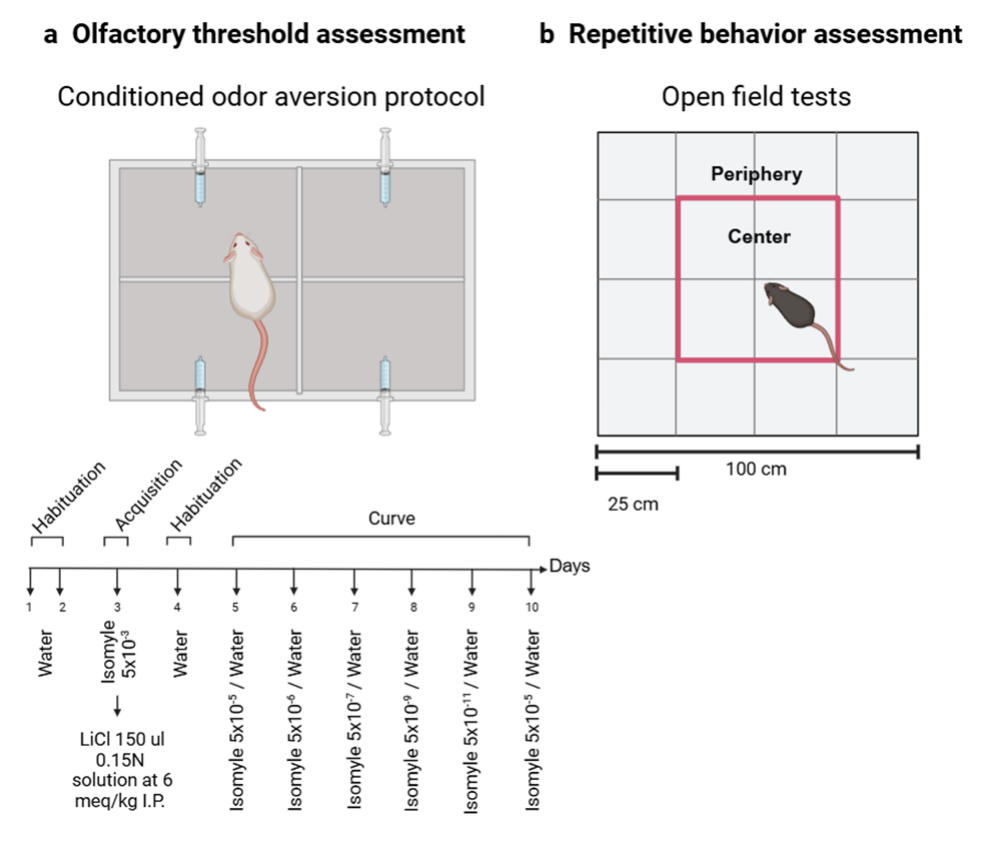
Behavior assessment method. **a** COA and olfactory threshold assessment. The di-agram shows the test-cage divided into 4 quadrants, in each of which a pipette was placed. Below is depicted the experimental design (habituation, acquisition, and assessment curve for olfactory threshold). **b** Spontaneous behavior assessment. The open-field arena was di-vided into 16 squares of 25 X 25 cm, delimiting the periphery and center areas

To evaluate olfactory system functioning, an odor detection acuity test was designed based on the conditioned odor aversion (COA) task. Mice were deprived of water for 16 h and submitted to a standard retronasal COA training paradigm for five consecutive days in which they were allowed to drink tap water or an odorized tasteless solution of isoamyl acetate (IA, artificial banana scent, SACF, St. Louis, USA) for 10 min as follows: on Days 1 and 2 (habituation), mice were placed in the experimental box and allowed to drink water from four 1 ml pipettes. On Day 3 (acquisition) an IA solution (5 × 10^–3^ M) was placed in all four pipettes; mice drank this solution and immediately afterward received an intraperitoneal LiCl injection. Each mouse was administered with approximately 150 ul of LiCl 0.15 M solution at 6 meq/kg dose, 2% BW (Risinger and Cunningham 2000; Rowland et al. 2004). On Day 4 (recovery), mice were allowed to drink water. Finally, from Day 5 to Day 10, they were tested for aversion learning permitting them to choose between decreasing concentrations of IA and water as follows: Day 5: 5 × 10^–5^%; Day 6: 5 × 10^–6^%; Day 7: 5 × 10^–7^%; Day 8: 5 × 10^–9^%; Day 9: 5 × 10^–11^%. To verify that the increase in AI consumption was not due to an odor aversion extinction effect, but rather to the inability to detect the odor due to the gradual decrease in its concentration, on the 10th day we offered again the AI 5 × 10^–5^% concentration. Water and IA-containing pipettes were located on opposite sides of the box and alternated between the trials to avoid place preference (Fig. 1a).

Each test was recorded using Anymaze software. After the test concluded, the consumption of each pipette was measured, including the IA solution, water, and total consumption.

#### Spontaneous behavior assessment

To study locomotor activity, repetitive behavior, and anxiety, we analyzed the spontaneous behavior through an open-field test. We used a square arena of 100 X 100 X 38 cm made of acrylic in which we recorded the frequency, time, and location of all locomotor activity during 5 min (300 s) (Brooks and Dunnett 2009). For anxiety assessment, the field was divided into two different areas, the center (50 X 50 cm) and the periphery, as shown in Fig. 1b.

Each trial was video-recorded using Any-maze software video tracking system version 4.73. The amount of feces and urine depositions for each mouse were also quantified.

### Statistical analysis

Normality was verified for each set of behaviors and brain structures; the data were subjected to a Shapiro–Wilk normality test. Since most of the data presented non-normal behavior, non-parametric statistics were used. To compare the metabolic activity of each structure between the strains and the behavior among strains, the Mann–Whitney test was used. The alpha level was set at *p* < 0.05.

## Results

### MicroPET metabolic uptake comparison between strain

**Fig. 2:**
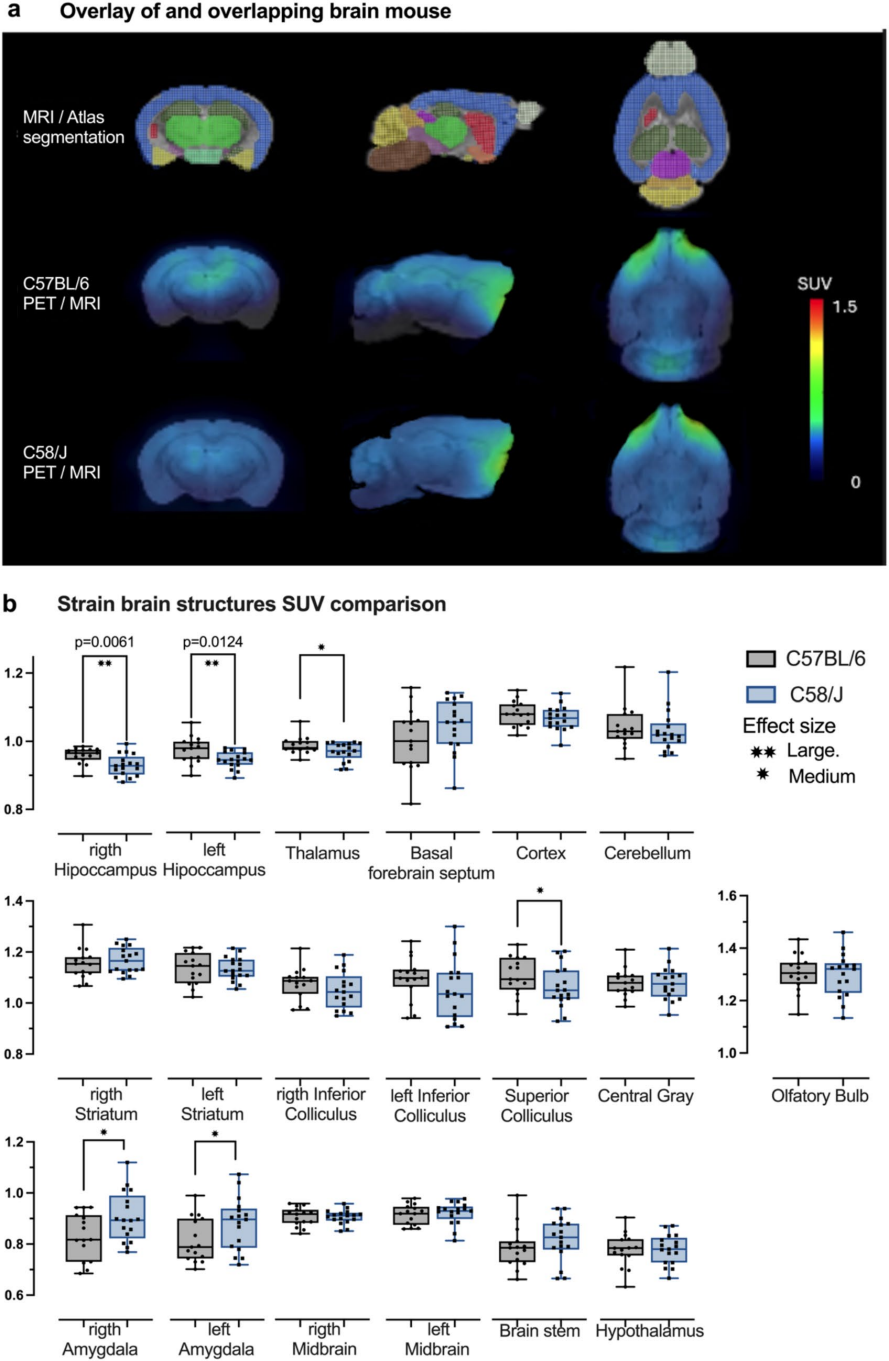
In **a** the first row shows a representative image of the overlay of an overlapping brain mouse to MRI and the M. Mirrione atlas segmentation. In the second row, the [^18^F]FDG microPET images are shown in coronal, sagittal and transversal sections of a brain characteristic of a C57BL/6 mouse, while in the last row a brain characteristic of a C58/J mouse is shown. In both, the hippocampus is highlighted for the green shaded area mainly in coronal view. **b** shows the comparison between both strains for each SUV brain structure. In all cases the box extends from the 2.5th to the 97.5th percentiles, the whiskers go down to the smallest value and up to the largest, and the middle line is plotted at the media. Each value is shown by a circle for the C57BL/6 and a square for the C58/J, the gray box represents the C57BL/6 and the blue box represents the C58/J. 21 C58/J mice and 19 C57BL/6 ones were used. To evaluate differences between strains, Mann-Whitey tests were used- the significant p value is shown in each graph- and the effect size was calculated using rank-biserial correlation

The SUV uptake was assessed in all brain structures, which were compared C58/J versus C57BL/6. The Mann–Whitney showed only significant differences between the right and left hippocampus; the SUV C57BL/6 was 0.965 and 0.9756 for right and left, respectively, while the SUV C58/J was 0.928 and 0.9414 lower than the SUV C57BL/6 J, as shown in Figs. 2a, b.

### Metabolic network differences

#### Correlation matrix

**Fig. 3:**
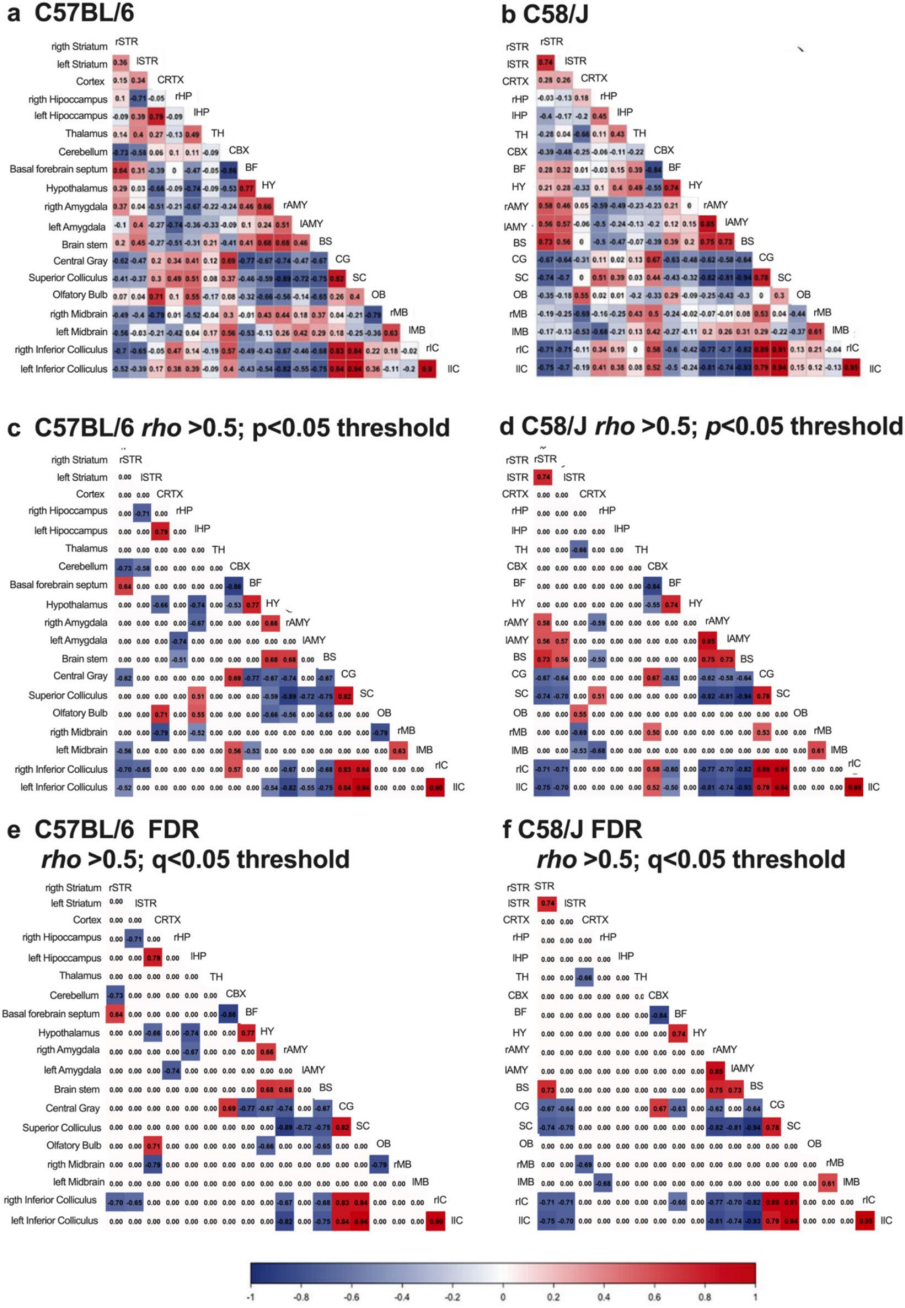
Correlation matrix. ROI × ROI Spearman correlation matrices of regional [^18^F]FDG uptake across subjects are shown for each strain. **a** for C57BL/6 and **b** for C58/J display the full correlation matrices, including positive and negative associations. Each cell shows Spearman’s ρ between ROI pairs; the color bar encodes sign and magnitude (blue = negative, red = positive; intensity =|ρ|). **c**, **d** are the matrices after applying a dual threshold (|ρ|> 0.5 and two tailed p < 0.05) for C57BL/6 and C58/J, respectively. **e**, **f** are the final adjacency matrices after controlling the false discovery rate (Benjamini–Hochberg); only edges meeting q < 0.05 and |ρ|> 0.5 are retained (**e** C57BL/6; **f** C58/J). Sample sizes for the C58/J and C57BL/6 groups were 21 and 19, respectively

For each strain, we computed an ROI × ROI Spearman correlation matrix across subjects from the regional SUV/SUVR values to construct the metabolic network. The full correlation matrices (including positive and negative associations) are shown in Fig. 3a (C57BL/6) and Fig. 3b (C58/J). We then applied a two-step threshold: magnitude (|ρ|> 0.5) and significance (two-tailed p < 0.05), yielding the filtered matrices in Fig. 3c–d. To control for multiple testing, p-values were adjusted using false discovery rate (Benjamini–Hochberg), and only edges meeting both criteria (q < 0.05 and |ρ|> 0.5) were retained; the final adjacency matrices are shown in Fig. 3e–f. As expected, FDR correction reduced the number of surviving connections.

#### Metabolic network

For each strain we constructed ROI × ROI Spearman correlation matrices across subjects (Fig. 3a-b). We then derived two adjacency matrices per strain: an uncorrected matrix retaining edges with |ρ|> 0.5 and two-tailed p < 0.05 (Fig. 3c–d), and a final FDR-corrected matrix in which edges additionally satisfied q < 0.05 (Benjamini–Hochberg; Fig. 3e–f). As expected, FDR correction reduced the number of surviving connections.

Graph-theoretical metrics were computed on these adjacency matrices (binary for degree, clustering, efficiencies, betweenness, modularity; absolute ρ used only for mean strength).

In the FDR-corrected networks, both strains had the same edge count (38; density = 0.222). As a sensitivity check, we repeated all analyses after exactly matching density (removing the weakest-|ρ| edge when needed); results were qualitatively unchanged (see Supplementary Table 1), so we report the metrics from the FDR- and magnitude-thresholded networks.

**Fig. 4:**
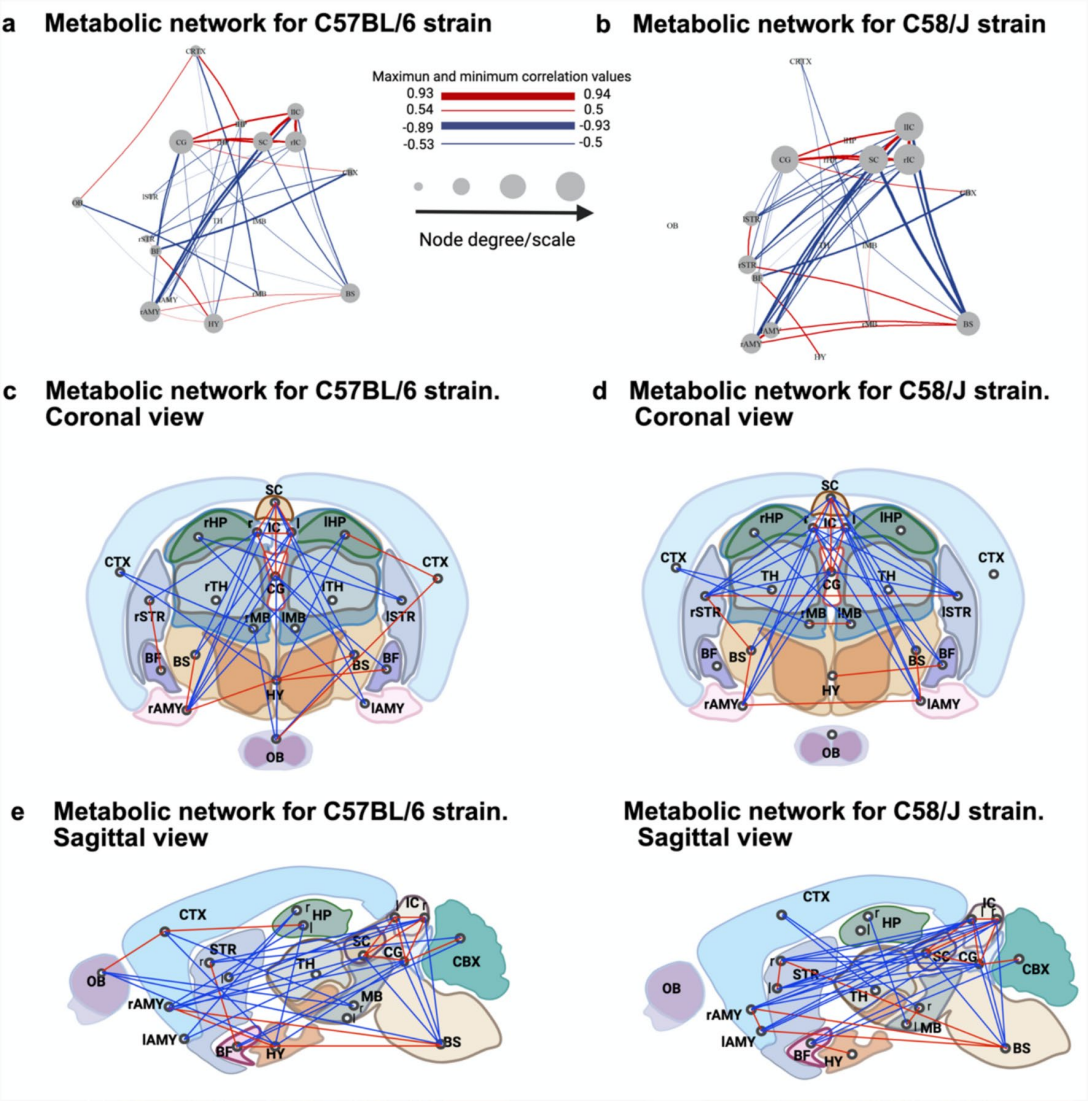
Metabolic brain networks by strain. In **a** for C57BL/6 and in **b** for C58/J metabolic correlation networks derived from ROI × ROI Spearman associations. Only edges surviving the dual threshold (|ρ|> 0.5 and FDR adjusted q < 0.05) are displayed. Edge color encodes sign (blue = negative, red = positive), and thickness scales with |ρ|. Node size reflects degree (number of connections). **c**, **d** are the coronal views highlighting inter hemispheric links for C57BL/6 and C58/J, respectively. **e**, **f** are the sagittal views emphasizing long range anterior–posterior connections for C57BL/6 and C58/J, respectively

Network visualizations for the final graphs are shown in Fig. 4a–b; Fig. 4c–d highlight inter-hemispheric connections. A small number of negative edges (|ρ|> 0.5) remained in C58/J, notably between the left and right striatum and the left and right inferior colliculus. Regionally, olfactory bulb (OB), cortex (CTX), and hypothalamus (HY) showed reduced connection density in C58/J relative to C57BL/6 (compare Fig. 3b *versus* 3f).

#### Modularity

**Fig. 5:**
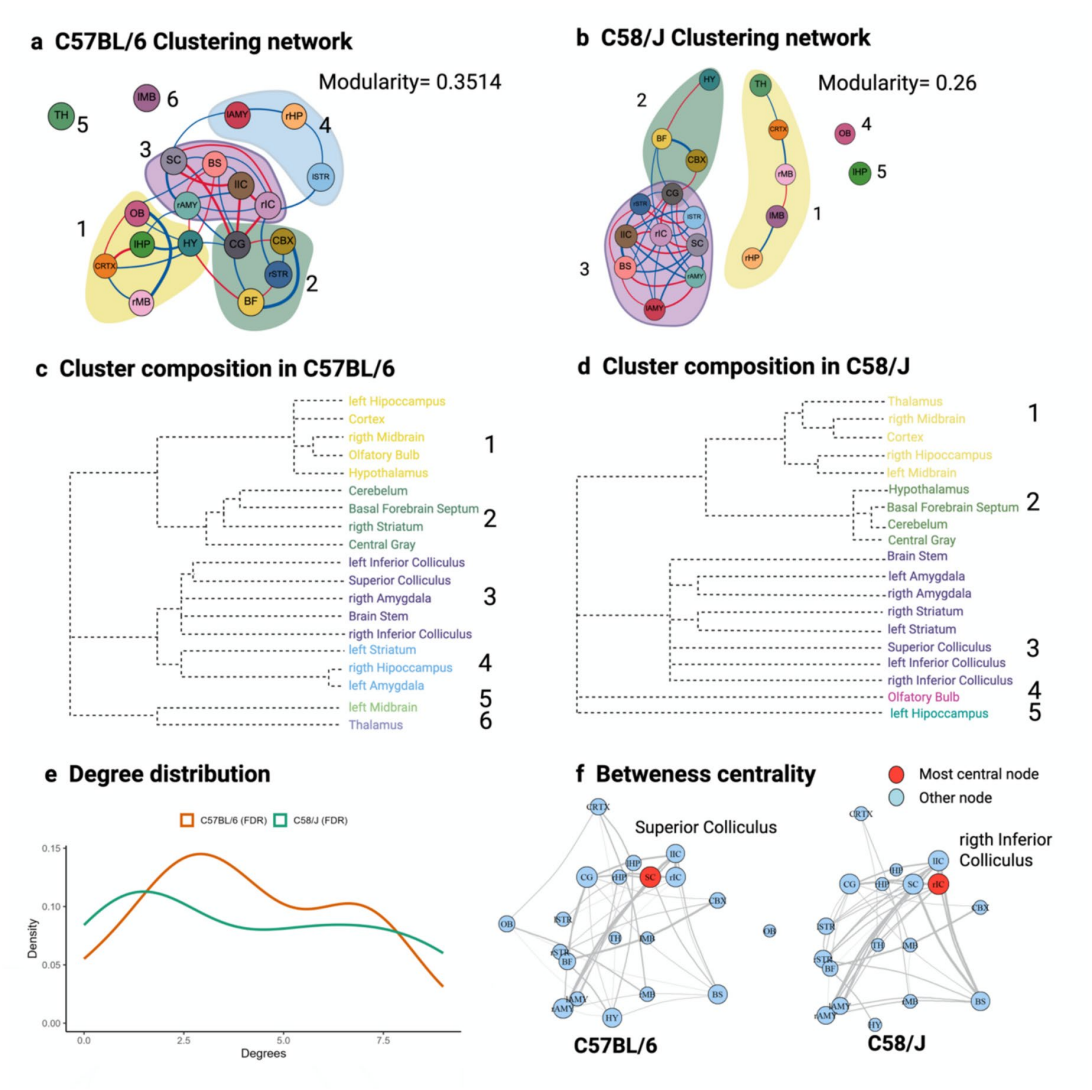
Strain brain network metrics. **a**, **b**, Cluster assignments and composition for the C57BL/6 and C58/J networks, respectively; the modularity (Q) value is shown in each panel. Nodes denote brain regions, and clusters are numerically labeled. **c**, **d** is the cluster composition together with the hierarchical clustering dendrogram for C57BL/6 and C58/J. **e** shown the degree distributions for both networks (green, C57BL/6; orange, C58/J). f shown the betweenness centrality, highlighting the most central node (in red) relative to the remaining nodes. Abbreviations: *lSTR/rSTR* Left/right striatum, *lHP/rHP* Left/right hippocampus, *lAMY/rAMY* left/right amygdala, *lIC/rIC* left/right inferior colliculus, *lMB/rMB* left/right midbrain, *CTX* cortex, *TH* thalamus, *CBX* cerebellum, *BFS* basal forebrain septum, *CG* central gray, *OB* olfactory bulb, *SC* superior colliculus, *BS* brainstem, *HY* hypothalamus posterior connections for C57BL/6 and C58/J, respectively.

Using the fast greedy method (see Supplementary Table 2 Newman and Girvan 2004; Newman 2004)), we identified four clusters with a modularity of 0.3514 in the C57BL/6 network and three clusters with a modularity of 0.2622 in the C58/J network, indicating different vertex connection densities. Figure 5 illustrates these clusters for each strain (a-b). In C58/J, the third cluster is more densely connected and is composed of nodes corresponding to clusters 3 and 4 of the C57BL/6 network, where the clusters exhibit more uniform connection densities. In C57BL/6 we identified four clusters: C1 (left hippocampus, cortex, right midbrain, olfactory bulb, hypothalamus); C2 (cerebellum, basal forebrain septum, right striatum, central gray); C3 (bilateral inferior colliculi, superior colliculus, right amygdala, brainstem); C4 (left striatum, right hippocampus, left amygdala). The thalamus and left midbrain were singletons, as shown in Fig. 5c. In C58/J, the cortex-containing cluster comprised the cortex plus right hippocampus, bilateral midbrain, and thalamus, while the left hippocampus and olfactory bulb were singletons. Cluster 2 mirrored C57BL/6’s C2, except the hypothalamus replaced the right striatum, which shifted to Cluster 3. This third cluster pooled nodes that, in C57BL/6, belonged to C3 and C4. Notably, the thalamus and left midbrain—singletons in C57BL/6—were embedded in Cluster 1 in C58/J as shown in Fig. 5d.

#### Degree distribution

The degree distribution (Boccaletti et al. 2006) in both networks is bimodal. While no significant difference in this metric was observed, the kurtosis shows a notable difference, being negative for the C58/J network (Fig. 5e). This indicates a greater dispersion of connection density (see Supplementary Table 2) in the C58/J network, whereas the C57BL/6 network exhibits a lower connection dispersion.

#### Betweenness centrality

With density matched (19 nodes, 38 edges per graph), mean weighted betweenness was markedly lower in C58/J than in C57BL/6 (0.018 vs 0.054; in supplementary Table 1), indicating fewer topological bottlenecks and a more distributed shortest-path topology in the covariance graph in C58/J (see Betweenness centrality means in Supplementary Table 2 (Newman 2010)). At the node level, the principal hub shifted from the superior colliculus in C57BL/6 to the right inferior colliculus in C58/J (centrality maps in the Fig. 5f).

#### Efficiency

**Fig. 6:**
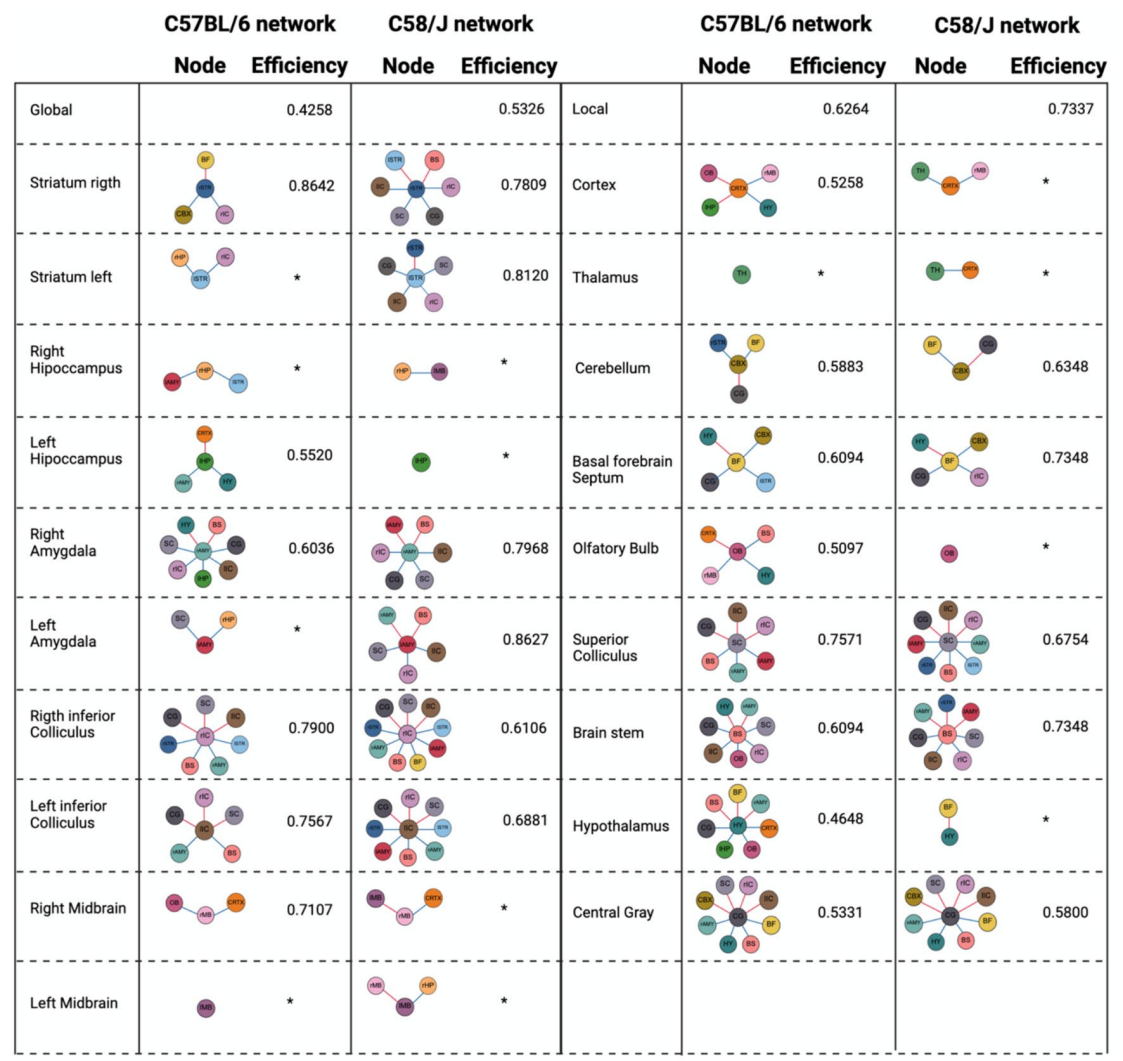
Network efficiency by strain. The top row reports global efficiency for each network. In the subsequent rows, the left column (C57BL/6) and right column (C58/J) display nodal (local) efficiency for each region together with a schematic of that node’s incident edges. Edges are colored by sign (blue = negative ρ, red = postive ρ); only connections surviving the dual threshold (|ρ|> 0.5 and FDR adjusted q < 0.05) are shown. An asterisk (*) marks nodes with minimal efficiency within their respective network

Global efficiency (Latora and Marchiori 2001) (see Supplementary Table 2) was 0.4258 for the C57BL/6 network and 0.5326 for the C58/J network (Fig. 6). The striatum and amygdala showed increased edge density and efficiency (see Local Efficiency in Supplementary Table 2) in C58/J compared to C57BL/6. Conversely, the left hippocampus, olfactory bulb, and hypothalamus had reduced connectivity and efficiency in the C58/J network (Fig. 6).

### Behavior assessment

For locomotor, repetitive and anxiety-like behavior, twenty-five C58/J mice were used and thirty C57BL/6 ones. These groups were independent of the groups used for micro-PET acquisition.

#### Olfactory threshold

**Fig. 7:**
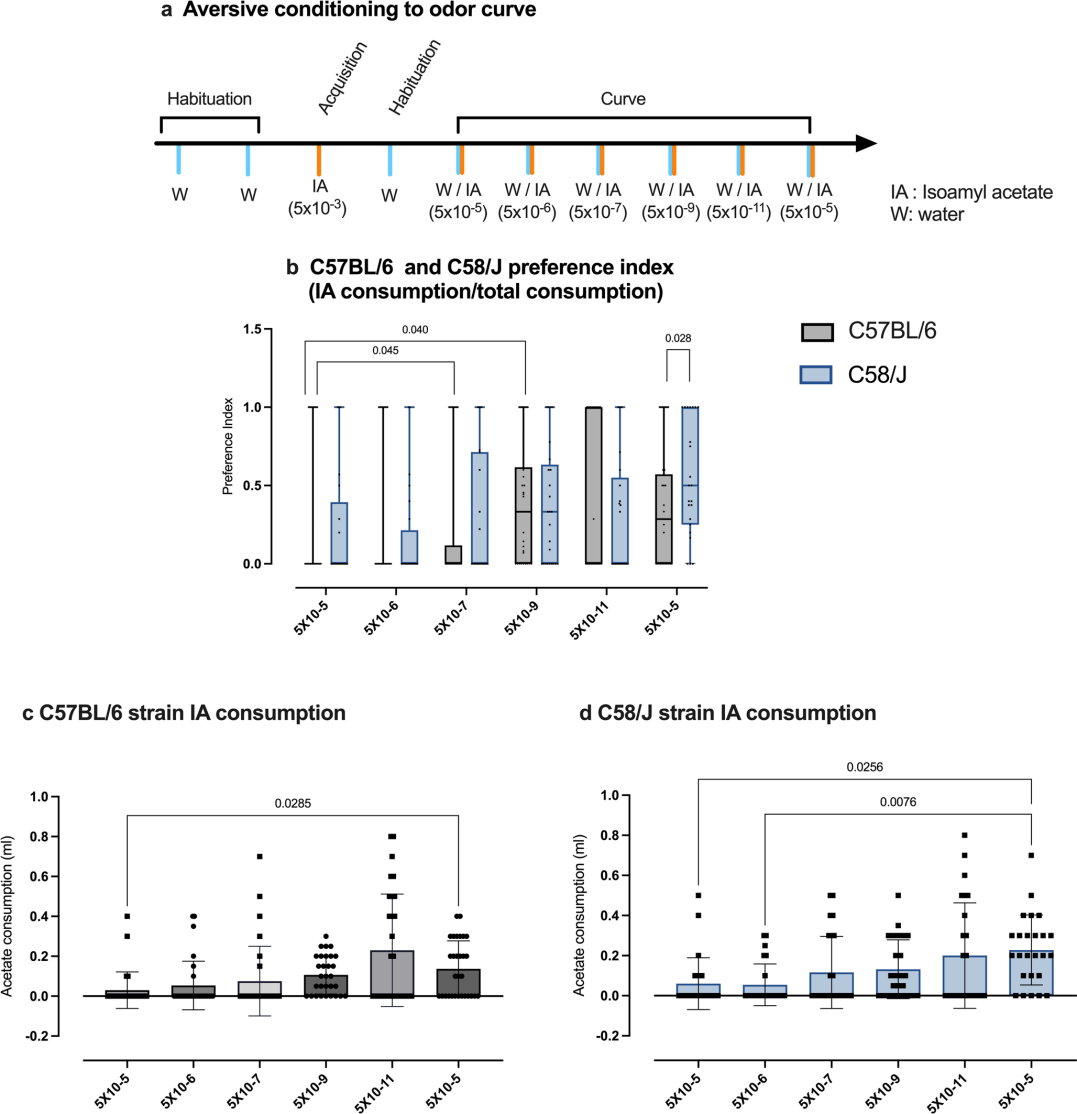
Olfactory function assessment. **a** experimental design showing the habituation days, COA acquisition, and the olfactory detection curve to IA. In **b** IA preference index (AI consumption/Total consumption) in both strains; C57BL/6 is shown in grey bars and C58/J in blue bars. **c** and **d**, showing IA consumption for the C57BL/6 and C58/J strains, respectively. Data are expressed as the Mean ± SEM in all cases. Sample size was twenty-nine for the C57BL/6 strain and twenty-five for C58/J strain. PI comparison between strains for each concentration was made with Mann–Whitney test; the comparison between IA concentrations was made by Friedman and Dunn´s correction. Sample sizes for the C58/J and C57BL/6 groups were 25 and 30 respectively

We evaluated odor perception by measuring the consumption of odorless water versus decreasing concentrations of IA solutions to which mice had been aversively conditioned two days before. Olfactory acuity was evaluated simultaneously in both strains, in five consecutive days as shown in the Fig. 7a. As shown in Fig. 7c and d, consumption of the more concentrated IA solution (5 × 10⁻^5^) was extremely low in both strains, indicating an adequate COA learning (Fig. 7b). Moreover, consumption patterns revealed that both C57BL/6 and C58/J avoided the IA solution at concentrations of 5 × 10⁻^5^, 5 × 10⁻⁶, and 5 × 10⁻⁷ during the first three days, indicating effective detection of the odor. However, at the fourth day, a significant increase in consumption of IA solution (5 × 10⁻⁹) was observed, suggesting that the mice could no longer discriminate accurately between the odorless water and the highly diluted IA solution, finally showing the highest consumption at the most diluted concentration (5 × 10⁻^11^). When mice were retested with the most concentrated testing solution of 5 × 10⁻^5^ on the sixth day, IA consumption significantly decreased compared to the most diluted concentrations (5 × 10⁻⁹ M and 5 × 10⁻^11^ M), indicating that aversive memory remained almost intact, but this response was only observed for the C57BL/6 strain (Fig. 7c).

In contrast, when the C58/J mice were choosing again between water and the most concentrated solution (5 × 10⁻^5^), IA consumption did not decrease but, on the contrary, it increased, suggesting an aversive memory extinction process. This pattern indicates an erratic response to aversive conditioning in C58/J mice (Fig. 7d).

#### Spontaneous behavior

**Fig. 8:**
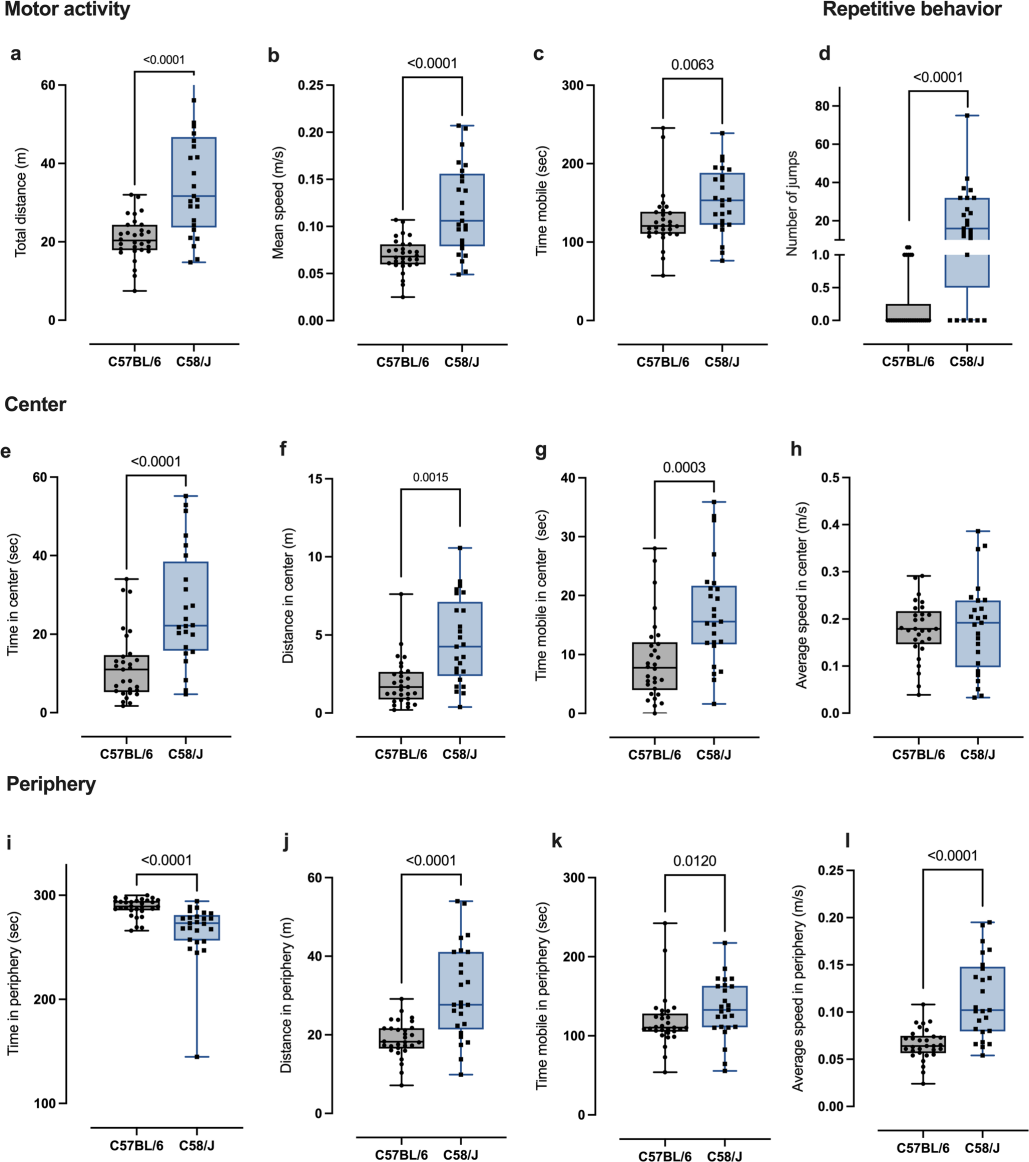
Spontaneous behavior assessment. **a**–**c** panels show the three motor activity components evaluated: total distance traveled, mean speed, and mobile time, respectively. **d** depicts the number of jumps across the entire arena. Panels **e** and **i** show the total time spent in the center and periphery zones, respectively. Panels **f**–**h** show the three locomotor activity components evaluated in the central zone, and panels **j**–**l** in the periphery. In all box-whisker graphs, the box extends from the 2.5th to the 97.5th percentiles, the whiskers go down to the lowest value and up to the highest, and the middle line is plotted at the median. Comparisons between strains for each indicator were made using the Mann–Whitney test. Sample sizes for the C58/J and C57BL/6 groups were 25 and 30 respectively

In animal models of ASD, abnormal or repetitive motor patterns have been considered analogous to stereotyped movements that are part of the diagnostic criteria for the human condition. To analyze these abnormal behavioral patterns, we evaluated three components of spontaneous horizontal motor activity, namely, the distance traveled in the open field, the average speed, and the total mobility time in five minutes (300 s). The number of jumps were recorded as a repetitive behavior index. The C58/J strain exhibited higher values than the C57/BL6 for all three components, as shown in Fig. 8a–c. Repetitive behavior was observed only in C58/J mice (Fig. 8d).

The three components were also evaluated in the periphery and center zones. Notably, the C58/J mice spend more time in the center than C57BL/6 mice (Fig. 8i). However, in both zones, the center (Figs. 8f–h) and in the periphery (Figs. 8i–k), the C58/J horizontal motor activity measured in distance, average speed, and mobile time remains higher than that of the C57BL/6 strain.

## Discussion

This study aimed to assess whether the C58/J mouse strain replicates some characteristics observed in ASD evaluated through [^18^F]FDG -PET neuroimaging and on the metabolic connectivity brain.

The neuroimaging findings obtained from [^18^F]FDG-microPET indicate that the C58/J strain exhibits decreased metabolic activity in the hippocampus compared with the C57BL/6 strain. A previous study reported a reduction in mature dendritic spines in the hippocampus of adult C58/J mice (Barón-Mendoza et al. 2021), which could be related to the hypometabolic activity observed in our study. Comparatively, has been observed a hippocampal hypometabolism in individuals with ASD (Sharma et al. 2018). Furthermore, Bauman and Kemper in 2005(Bauman and Kemper 2005) identified reduced neuronal size and increased cell packing density in the hippocampus of autistic individuals, suggesting early developmental disruptions that may compromise hippocampal structure and function. Collectively, these findings underscore the pivotal role of hippocampal abnormalities in ASD pathophysiology, implicating both early structural changes and ongoing metabolic dysfunction.

The brain’s organization supports the processes of cognition, behavior, and perception (Park and Friston 2013). Recent years has been studied through network theory that describes connectivity phenomena. Functional connectivity derived from fMRI has been extensively characterized in ASD, whereas metabolic connectivity from [^18^F]FDG-PET is comparatively less explored. The fMRI BOLD signal reflects hemodynamic fluctuations linked to neural activity, offering millimeter-scale spatial and second-scale temporal resolution while [^18^F]FDG-microPET quantifies regional glucose uptake and phosphorylation (hexokinase-mediated), indexing synaptic energy demand integrated over tens of minutes (Phelps et al. 1979; Millevert et al. 2023).

Accordingly, metabolic network organization in static [^18^F]FDG-PET can be characterized by quantifying interregional covariation in [^18^F]FDG uptake across individuals scanned under the same experimental context (Sala and Perani 2019; Millevert et al. 2023). Because each scan provides a single, uptake-period–integrated estimate per region for each subject, the resulting covariance network constitutes a group-level representation that is specific to the shared condition or state present during the acquisition window (e.g., task or disease) in the study group (Sala and Perani 2019; Millevert et al. 2023).

Regarding metabolic connectivity in ASD, the only study conducted by Lee et al. in 2012, which proposed a metric to evaluate internodal distance in [^18^F]FDG-PET recoding of children with ASD, ADHD, and healthy controls, found that the connections (links) distribution plot of the metabolic network in individuals with ASD exhibited a heavy tail and a sharp peak. This topology suggests simultaneous local overconnectivity (evidenced by the peak of the curve) and global underconnectivity (evidenced by the long, broad tails) (Lee et al. 2012).

Compared to the control population, ASD metabolic networks displayed weaker connections between the left inferior prefrontal regions and other brain areas; the authors posit that this topology may be related to aspects of ASD symptomatology (Lee et al. 2012). Although metabolic covariance networks and fMRI-based functional networks index different processes, convergent topological alterations across modalities have been reported when these findings are interpreted at the appropriate level of inference (Dapretto et al. 2006; Courchesne et al. 2007). In the same study, ADHD metabolic networks showed reduced connections between the sensorimotor region and various frontoparietal regions, including the anterior cingulate cortex; this hypoconnectivity could explain deficits in attentional cognitive control and sensorimotor integration in this condition (Piek and Dyck 2004). Together, these observations suggest that FDG-PET covariance networks can provide complementary network-level information alongside functional and structural data, while recognizing that any cognition–network associations inferred from covariance networks are indirect and should be interpreted cautiously (Dapretto et al. 2006; Courchesne et al. 2007).

However, there is evidence demonstrating that metabolic networks share partial concordance with functional and structural networks (Savio et al. 2017; Millevert et al. 2023; Tuan et al. 2026). Across modalities, graph-theoretical descriptions often converge on canonical topological features—such as small-world organization—in healthy individuals and can reveal network-level disruptions in neurological disease (Savio et al. 2017; Tuan et al. 2026). These convergences support cross-modal comparisons at the level of network topology, while acknowledging that each modality reflects distinct physiological processes and therefore does not have the same interpretative meaning (Sala et al. 2023; Millevert et al. 2023). Consequently, FDG-PET–derived network measures still require continued validation across tasks, cognitive processes, and specific conditions, analogous to the validation that BOLD-based measures have undergone in recent years.

Given the marked differences between functional and metabolic connectivity, the present work asks whether the C58/J strain exhibits a group-level [^18^F]FDG metabolic covariance network organization that is qualitatively consistent with FDG-PET network alterations reported in ASD. With this objective, we operationalized metabolic connectivity as statistical dependence in [^18^F]FDG uptake between brain regions across individuals from static acquisitions (see Methods). The resulting ROI × ROI metabolic covariance networks provide a complementary, trait-level view of organization—reflecting coordinated regional energetic demand across subjects—rather than the moment-to-moment temporal synchrony captured by fMRI. Because our atlas used a coarse parcellation (19 ROIs) including a composite ‘cortex’ node, all graph-theoretical findings should be interpreted at a macroscale and do not resolve fine-grained intra-cortical organization.

The C58/J group-level metabolic covariance network showed reduced edge density among regions implicated in social interaction and sensory processing, including the olfactory bulb, hippocampus, and hypothalamus. In contrast, motor control regions—such as the striatum, superior and inferior colliculi, and, left amygdala—were more densely connected (i.e., showed more suprathreshold correlations in magnitude) and exhibited higher graph-theoretical efficiency metrics. Behaviorally, C58/J mice displayed no deficits in olfactory threshold but showed rapid extinction of COA, consistent with impaired memory retention, along with heightened repetitive motor activity. At the level of network topology, reduced connectivity between the olfactory bulb and limbic regions such as the hippocampus, hypothalamus, and, amygdala (Fig. 5) is broadly consistent with reports of altered metabolic network organization in ASD and suggests disrupted integration of olfactory information. Olfactory cues are essential for social communication, mating behaviors, and territorial marking, among many other functions (Mori and Sakano 2011; Sokolowski and Corbin 2012). Although odor detection acuity appears to be preserved, altered processing of socially relevant odor cues may contribute to the social deficits observed in C58/J mice and may reflect sensory processing abnormalities (Dudas et al. 2024).

Our behavioral assessments confirm that C58/J mice display increased locomotor activity and repetitive movements, consistent with the motor stereotypies characteristic of ASD (Ryan et al. 2010; Muehlmann et al. 2012).

The C58/J metabolic network exhibited comparatively higher connectivity and graph-theoretical efficiency in motor control regions, including the striatum. This strain-level network pattern co-occurs with the behavioral phenotype of increased repetitive and stereotyped movements observed in C58/J mice and is consistent with the established role of the striatum in motor function and habit formation. In ASD, striatal alterations—including reports of increased functional connectivity—have been associated with repetitive behaviors (Langen et al. 2009), and striatal volume/connectivity measures have been reported to correlate with repetitive behavior severity (Estes et al. 2011; Hunnicutt et al. 2016).

In contrast, C58/J mice showed reduced metabolic covariance involving the hypothalamus, pointing to region-specific network alterations rather than a global increase in connectivity. The hypothalamus regulates behaviors such as social interactions, feeling responses, and homeostasis maintenance (Saper and Lowell 2014). Studies have reported structural abnormalities in the hypothalamus of individuals with ASD, including reduced gray matter volume and altered functional connectivity (Wolfe et al. 2015). Dysfunction in hypothalamic circuits may contribute to the social and emotional deficits observed in ASD (Caria and Dall’Ò 2022). In this context, reduced hypothalamic connectivity in C58/J may be relevant to the social interaction impairments reported in this strain.

Interestingly, the global efficiency of the FDG metabolic covariance network was higher in the C58/J strain compared to controls. In graph-theoretical terms, higher global efficiency corresponds to shorter average path length within the covariance graph (i.e., more globally distributed covariance), and should not be interpreted as enhanced within-subject communication. One possibility is that this reflects a topology that combines locally concentrated covariance with altered long-range covariance, which could be conceptually related (at the level of graph topology) to the local overconnectivity/long-range underconnectivity framework proposed in ASD functional connectivity studies (Belmonte et al. 2004; Supekar et al. 2013; Keown et al. 2017). However, because modalities differ and our networks are group-level covariance estimates, these comparisons remain interpretive and require further validation.

Our findings indicated some deficits in odor aversion memory maintenance in C58/J mice. While olfactory threshold assessment showed that these mice not only respond adequately to COA, but also that their olfactory acuity is well preserved, they seem to extinguish conditioning faster than the control strain. Therefore, the impairments in social interaction and other highly olfactory-dependent behaviors in the C58/J strain might not be due to a sensory deficiency in olfactory detection but rather to the significance of the repertoire of odors they have to deal with in order to respond appropriately. Sensory sensitivities and atypical responses to sensory stimuli are common in ASD and can affect social interactions and environmental engagement (Ji et al. 2026).

Finally, several limitations should be acknowledged. First, because the metabolic network was derived from between-subject covariance of static [^18^F]FDG uptake, it does not reflect within-subject co-variation dynamics as functional networks can; accordingly, inferences are restricted to the group-level covariance organization captured by the strain-level network estimate.

Second, a limitation of the present graph analysis is the coarse parcellation (19 ROIs), including a composite ‘cortex’ node. Graph measures depend on network size and node definition/parcellation scale; therefore, the obtained values characterize macroscale metabolic covariance organization at this resolution and do not permit inference about fine-grained intra-cortical topology or cortical subregional hubs. This choice reflects a trade-off between anatomical specificity and quantification reliability in mouse microPET, where limited spatial resolution and partial volume effects can bias uptake estimates and interregional correlations for small parcels.

Third, our study included only male mice. This decision was driven by the strong male bias observed in the clinical population, where ASD diagnosis is approximately four times more prevalent in males than in females (Loomes et al. 2017; Maenner 2021). While previous characterizations of the C58/J strain have reported that social deficits and repetitive behaviors are present in both sexes (Dudas et al. 2024), recent evidence suggests that distinct molecular or hormonal mechanisms may underlie ASD-like phenotypes in males versus females (Loomes et al. 2017; Maenner 2021). By restricting our analysis to males, we cannot rule out sex-specific protective mechanisms or differential sensitivity to the molecular pathways investigated herein. Future studies in our laboratory will specifically address this sexual dimorphism to determine if the metabolic network changes observed in C58/J males are conserved in female counterparts.

## Conclusions

The convergence of ASD-relevant behavioral phenotypes with a distinctive pattern of group-level [^18^F]FDG metabolic covariance network organization in the C58/J strain provides convergent support for this strain as a model to study ASD-relevant neurobiology. Overall, C58/J mice showed decreased hippocampal glucose metabolism, altered conditioned odor aversion (COA) extinction dynamics, and increased stereotyped motor behavior, together with a metabolic covariance network characterized by relatively sparse olfactory bulb–hippocampus/hypothalamus covariance and denser covariance among motor-related regions.

Future work should extend these findings by (i) applying higher-resolution and/or complementary imaging approaches (e.g., fMRI or microscopy-based methods) to better resolve smaller nuclei and circuit-level organization, (ii) including female mice to examine sex-specific mechanisms and improve generalizability, and (iii) integrating molecular and genetic analyses (e.g., neurotransmitter systems, synaptic proteins, and gene expression profiles) to identify mechanistic drivers of the observed network alterations. These conclusions should be interpreted in light of the methodological limitations discussed above.

## Supplementary Information

Below is the link to the electronic supplementary material.

Supplementary file1 (DOCX 15 KB)

## Data Availability

The datasets used and/or analyzed during the current study are available from the corresponding author on reasonable request.
