# Supplementary material for: [18F]FDG metabolic brain network in C58/J strain: an autism murine model
Source: Brain Struct Funct. 2026 Mar 4;231(3):34. doi: 10.1007/s00429-026-03087-8 (PMC12960385; doi:10.1007/s00429-026-03087-8)
Supplement: Supplementary file 1 — Supplementary file1 (DOCX 15 KB) [file 429_2026_3087_MOESM1_ESM.docx]

**[^18^F]FDG Metabolic brain network in the C58/J mouse strain:**

**A murine model of autism**

**Supplementary tables**

Supplementary Table 1. Network metrics in corrected and no-corrected matrices

| Strain | Threshold | Edges | Density | Mean degree | Mean strength abs | Clustering bin | Global efficency | Local efficiency | Mean betweenness | Modularity |
| --- | --- | --- | --- | --- | --- | --- | --- | --- | --- | --- |
| C57BL/6 | p<0.05,  \|ρ\|>0.5 | 55 | 0.3216 | 5.7895 | 3.9737 | 0.5648 | 0.4434 | 0.6007 | 0.0427 | 0.2871 |
|  | ;  \|ρ\|>0.5; q<0.05 | 38 | 0.2222 | 4.0000 | 2.9789 | 0.5536 | 0.4258 | 0.6264 | 0.0540 | 0.3514 |
| C58/J | p<0.05,  rho> 0.5 | 55 | 0.3216 | 5.7895 | 4.0111 | 0.7480 | 0.4079 | 0.6664 | 0.0588 | 0.2331 |
|  | ;  \|ρ\|>0.5; q<0.05 | 38 | 0.2222 | 4.0000 | 3.0297 | 0.7727 | 0.5326 | 0.7337 | 0.0182 | 0.2622 |

Supplementary Table 2. Network metrics description

| **Metric** | **Description** | **Formula** | **Reference** |
| --- | --- | --- | --- |
| Modularity | Modularity measures how well a network can be divided into smaller groups called modules or communities. Each module has many connections within it but fewer connections with other modules. This helps identify different functional areas within a network. | $Q\text{=}\frac{1}{2m}\sum_{ij} \left[ A_{ij}\text{-}\frac{k_{i}k_{j}}{2m} \right]\delta\left( C_{i},C_{j} \right)\text{ }$ | Newman, M.E.J and Girvan, M, 2004 Finding and evaluating community structure in networks; Newman, M. E. J. 2004. Fast algorithm for detecting community structure in networks |
| Degree Distribution | Degree distribution shows how many connections each node (a point in the network) has. In a brain network, this can help identify which brain regions are highly connected (hubs) and which are less connected. | $k_{i}\text{=}\sum_{j\in G} a_{ij}$ | Boccaletti, S., Latora, V., Moreno, Y., Chavez, M., & Hwang, D. U. Complex networks: Structure and dynamics (2006). |
| Betweenness Centrality | A topological centrality metric quantifying how frequently a node lies on shortest paths between other nodes in the network. In metabolic covariance networks, betweenness centrality reflects the organizational role of a region within the pattern of shared metabolic variance across the graph. | $B_{i}\text{=}\sum_{m\neq\in\in G} \frac{\sigma_{mn}\left( i \right)}{\sigma_{mn}}$ | Newman, M. E. J. Networks: An Introduction (2010) |
| Global Efficiency | The average inverse shortest path length across the network. In metabolic covariance networks, global efficiency summarizes the overall topological integration of shared metabolic variance among brain regions. | $E_{global}\left( G \right)\text{=}\frac{1}{N\left( N\text{-}1 \right)}\sum_{i\neq j\in G} \frac{1}{d_{ij}}$ | Latora, V., & Marchiori, M. Efficient Behavior of Small-World Networks (2001) |
| Local Efficiency | A measure of the efficiency of local subgraphs surrounding each node. In metabolic covariance networks, local efficiency reflects the degree of local clustering and segregation of shared metabolic variance. | $E_{local}\left( G \right)\text{=}\frac{1}{N}\sum_{i\in G} E_{global}\left( G_{i} \right)$ |  |
